# Inter-coat protein loading of active ingredients into *Tobacco mild green mosaic virus* through partial dissociation and reassembly of the virion

**DOI:** 10.1038/s41598-024-57200-0

**Published:** 2024-03-26

**Authors:** Ivonne González-Gamboa, Adam A. Caparco, Justin McCaskill, Paulina Fuenlabrada-Velázquez, Samuel S. Hays, Zhicheng Jin, Jesse V. Jokerst, Jonathan K. Pokorski, Nicole F. Steinmetz

**Affiliations:** 1 Department of NanoEngineering, University of California, San Diego, La Jolla, CA USA; 2 Department of Bioengineering, University of California, San Diego, La Jolla, CA USA; 3 Department of Radiology, University of California, San Diego, La Jolla, CA USA; 4 Center for Nano-ImmunoEngineering, University of California, San Diego, La Jolla, CA USA; 5 Institute for Materials Discovery and Design, University of California, San Diego, La Jolla, CA USA; 6 Moores Cancer Center, University of California, San Diego, La Jolla, CA USA; 7 Center for Engineering in Cancer, Institute of Engineering in Medicine, University of California, San Diego, La Jolla, CA USA; 8 Shu and K.C. Chien and Peter Farrell Collaboratory, University of California, San Diego, La Jolla, CA USA; 9 Materials Science and Engineering Program, University of California San Diego, 9500 Gilman Dr, La Jolla, CA USA; 10 Department of Molecular Biology, University of California, San Diego, La Jolla, CA USA

**Keywords:** Plant virus, Tobacco mild green mosaic virus, Pesticide nanocarriers, Drug delivery, Precision agriculture, Nanobiotechnology, Drug delivery

## Abstract

Chemical pesticide delivery is a fundamental aspect of agriculture. However, the extensive use of pesticides severely endangers the ecosystem because they accumulate on crops, in soil, as well as in drinking and groundwater. New frontiers in nano-engineering have opened the door for precision agriculture. We introduced Tobacco mild green mosaic virus (TMGMV) as a viable delivery platform with a high aspect ratio and favorable soil mobility. In this work, we assess the use of TMGMV as a chemical nanocarrier for agriculturally relevant cargo. While plant viruses are usually portrayed as rigid/solid structures, these are “dynamic materials,” and they “breathe” in solution in response to careful adjustment of pH or bathing media [e.g., addition of solvent such as dimethyl sulfoxide (DMSO)]. Through this process, coat proteins (CPs) partially dissociate leading to swelling of the nucleoprotein complexes—allowing for the infusion of active ingredients (AI), such as pesticides [e.g., fluopyram (FLP), clothianidin (CTD), rifampicin (RIF), and ivermectin (IVM)] into the macromolecular structure. We developed a “breathing” method that facilitates inter-coat protein cargo loading, resulting in up to  ~ 1000 AIs per virion. This is of significance since in the agricultural setting, there is a need to develop nanoparticle delivery strategies where the AI is not chemically altered, consequently avoiding the need for regulatory and registration processes of new compounds. This work highlights the potential of TMGMV as a pesticide nanocarrier in precision farming applications; the developed methods likely would be applicable to other protein-based nanoparticle systems.

## Introduction

Year after year, plant parasites burden the agricultural sector resulting in staggering crop losses. Numerous species of insects and worms, such as moths, beetles, and nematodes, have been identified as significantly harmful to plant species^1–3^. Current methods for controlling pest populations range from broad use of chemical insecticides to genetically modifying crops to increase their resistance to target species^4,5^. Pesticides have long been applied as a method for crop yield increase. These products, typically chemicals designed to either kill or discourage harmful pests, can be beneficial. While the broad specificity and quick deployment of chemical insecticides may make them more favorable than some genetic engineering options, numerous environmental and health concerns have risen. For instance, the extensive use of pesticides in agriculture causes these toxins to accumulate on crops, in soil, as well as in drinking and groundwater reserves, indicating runoff from their application site^6–8^. There is a need for a pesticide application system which maintains pesticide efficacy while reducing the environmentally harmful runoff. Nanotechnology formulations promise to package and deliver active ingredients to the target site, therefore increasing the therapeutic index and protecting the environment^9,10^.

Recently, viral nanoparticle (VNP) systems—in particular those from plant viruses and bacteriophages—have shown versatility in the delivery of target molecules, both in the medical and agronomic sector^11^. For agronomic applications, plant viruses can be rendered non-infectious to protect crops^12^ and for medical applications their immunogenicity can be harnessed for immunotherapy^13–15^ or balanced through appropriate coatings^16,17^. The macromolecular structures of viruses can be regarded as high molecular weight biopolymers. Their highly functionalizable inner and outer surfaces^18–20^, in combination with ease of manufacture at scale and excellent biological stability, make VNPs ideal delivery systems. In fact, the development pipeline is moving rapidly with various VNP systems being developed for targeted drug delivery^21–25^ as well as agrochemical delivery^11,12,26,27^. Our work has been centered on TMGMV, a rod-shaped plant virus measuring 300 × 18 nm; a “living” nanomaterial that has gained EPA-approval and is used as a bioherbicide in the state of Florida^28,29^. With a goal to transform TMGMV as a pesticide carrier system to target pests residing in soil, our lab has demonstrated that TMGMV has suitable soil mobility, while offering a versatile engineering design space for functionalization^27,30,31^. Like many VNPs, the bioconjugation sites of TMGMV have been categorized^19^. Covalent conjugation however is a complex and resource-intensive process, and pesticides are challenging to conjugate to proteins given their high degree of hydrophobicity^32^. Furthermore, the costs and regulatory processes associated with using covalent conjugation strategies for pesticide linking to VNPs (including classifying, characterizing, and approving covalently modified chemicals) outweigh the benefits for agricultural use. Thus, an efficient non-covalent method of loading VNPs is desired.

Extensive work in the space of physical virology has demonstrated that viruses are dynamic assemblies that respond to their environments and bathing conditions^33–36^. For example, previous work with Red clover necrotic mosaic virus (RCNMV) showed that the virus assembly is dynamic and changes in bathing condition (EDTA treatment at pH 7.5) allowed conformational pore opening allowing positively charged cargo to be gated into the capsid; the pore formation is a reversible process, therefore providing a means of trapping the AI^27^. Furthermore, extensive structural work has been conducted using Tobacco mosaic virus (TMV), a close relative of TMGMV. TMV rods are stable under physiologic conditions. At pH 7 the coat proteins of TMV form either so-called 20 s disks or lock-washer disks—the latter promoting rod assembly^37,38^. Exposing TMV to higher pH destabilizes the coat protein-coat protein interactions leading to rod or disk disassembly into lower unit assemblies (4S), dimers, and free coat proteins^39^. It is noteworthy that these processes are both dynamic and reversible^40^. Changes in pH result in changes in the ionization of protein residues, which in turn affect electrostatic interactions between them^41^. When the capsid proteins start to dissociate, the nanoparticle structure breathes and pores are opened allowing access to the inter-coat protein space. Besides pH, solvents also trigger assembly/disassembly—for example, DMSO at concentrations of up to 10% by volume has little effect on the structure of proteins^42^, while higher DMSO concentrations tend to destabilize the proteins leading to dissociation and unfolding, due to its effects on charge state distribution^43^, as well as disrupting structural water in the protein^44^. DMSO primarily disassembles TMV from the 5’ end, although part of the rods uncoat more slowly and less extensively from the 3’ end (the differences can be explained by the differences in RNA sequences at the 5’ vs 3’ end of the ss(+)RNA cargo)^45^.

**Figure 1:**
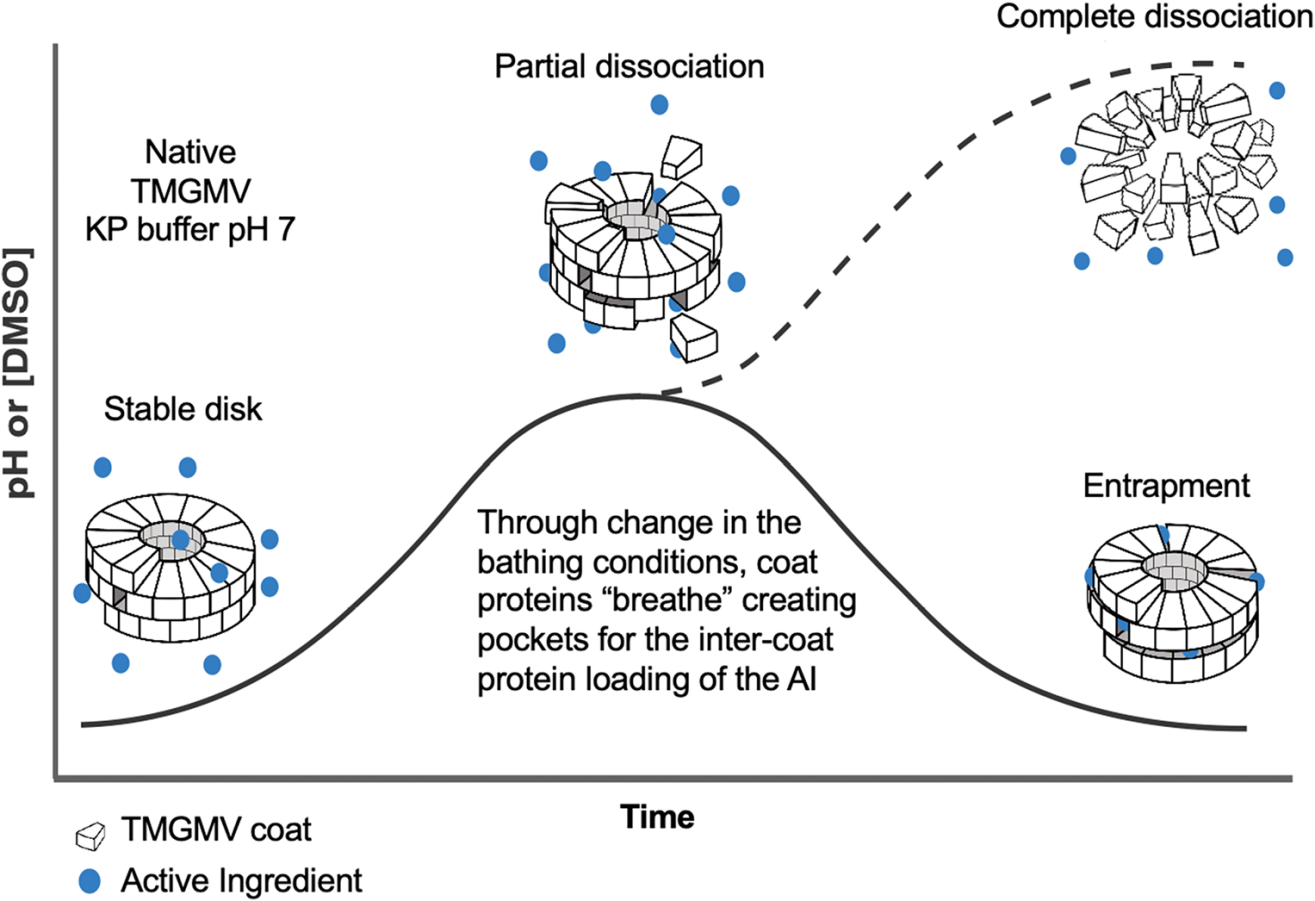
Schematic representation of the “breathing” phase transition diagram. AI = active ingredient.

Building on these prior works from the physical virology field, we set out to make use of these dynamic processes to load cargo for agricultural applications. We developed a “breathing” method for TMGMV based on careful pH adjustment or DMSO concentration and loaded molecules such as fluopyram, clothianidin, rifampicin and ivermectin (Fig. 1). The nanoparticle formulations and TMGMV structures were characterized by a combination of techniques to determine particle integrity, AI infusion, secondary structure stability post infusion.

## Materials and methods

### Preparation of TMGMV

TMGMV was obtained from BioProdex (Gainesville, FL, USA) and stored at − 20 °C. The solution was thawed at 4 °C overnight and then dialyzed against potassium phosphate buffer (KP; 10 mM, pH 7.2) for 24 h at 4 °C using 12–14 kDa dialysis tubing (Fisher Scientific S432700; Waltham, MA, USA). The buffer solution was replaced, and the dialysis continued for an additional 48 h. The solution was then centrifuged at 10,000 × g for 20 min (Beckman Coulter Allegra or Avanti centrifuges). Once the supernatant was collected and ultracentrifuged at 212604 × g for 2.5 h at 4 °C (Beckman Coulter Optima L-90 k Ultracentrifuge with 50.2 Ti rotor; Brea, CA, USA), the pellet was resuspended under rotational mixing overnight at 4 °C in KP buffer. TMGMV concentration was confirmed using a Nanodrop 2000 (Thermo Scientific; Waltham, MA, USA) and lastly, the concentration was adjusted to 10 mg mL^−1^ in 10 mM KP before storing at 4 °C (for TMGMV CP, ε_260_ = 3 mL mg^−1^ cm^−1^).

### Infusion of hydrophobic cargo in TMGMV by pH increase from pH 7 to pH 7.5

TMGMV at a concentration of 1 mg mL^−1^ in 10 mM KP buffer at pH 7.5 was kept for 10 days at 4 °C. The following AIs were used: fluopyram and clothianidin, (BASF, Berkeley, CA, USA), rifampicin and ivermectin (BioVision; Milpitas, CA, USA). Cyanine 5 (Cy5; Lumiprobe; Cockeysville, MD, USA) and doxorubicin (DOX; ApexBio; Houston, TX, USA) were also studied as proof of concept (fluorescent molecule and cancer chemotherapy). The AI was added to TMGMV by adding an excess of 10:1 AI:CP (each TMGMV rod is assembled from  ~ 2100 identical CPs) every day until a ratio of 100:1 was reached. During this process the reaction was kept mixing on a rotary shaker. 1 mL aliquots were obtained each day for further analysis.

After AI loading, the aliquots were spin filtered using 100 K molecular weight cut-off 0.5 mL filters (MilliporeSigma, Burlington, MA, USA). 200 μL of the aliquot and 250 μL of KP solution were added and then centrifuged at 16,160 × *g* for 5 min at 4 °C, the flow through was discarded, and then 450 μL of KP was added and centrifuged again, this step was repeated 3 times. After the third centrifugation, the filter was inverted in a new tube and centrifuged at 1000 × *g* for 2 min, to recover the supernatant and carry out the subsequent characterization.

### Infusion of hydrophobic cargo in TMGMV by change in DMSO concentration

TMGMV in 10 mM KP buffer (pH 7.2) was diluted to 5 mg mL^−1^ in 2 mL of buffer and transferred to a 25 mL beaker and magnetically stirred at 300 rpm at room temperature. A solution of DMSO and 10 mM KP was added dropwise to dilute the solution to a 20% (v/v) concentration of DMSO and 2 mg mL^−1^ of TMGMV. Aliquots of the AIs were added dropwise to the solution to prevent precipitation. The solutions were left to stir at room temperature for 24 h. The samples were collected and spin filtered as described above before being stored at 4 °C.

### Transmission electron microscopy (TEM)

TMGMV samples were diluted to a concentration of 0.05 mg mL^−1^ and absorbed onto carbon-coated TEM grids (Electron Microscopy Sciences, Hatfield, PA, USA). The grids were then washed three times with pure water. Then, grids were stained by 2% (w/v) uranyl acetate for 90 s. TEM was conducted using a FEI Tecnai F30 transmission electron microscope operated at 300 kV. Image analysis was performed using ImageJ software (https://imagej.nih.gov/ij/download.html). To determine the change in width of the nanoparticles, we measured the width of sections of 100 nm in length for standardization purposes. Thirty TMGMV particles in total per sample were analyzed. TMGMV particle length as well as the perimeter and the area were measured; these data were then used to calculate the average width from the perimeter. For statistical analysis, one-way Anova and Student’s T-test for independent populations were performed; all populations had a normal distribution (Shapiro Wilk test).

### Size exclusion chromatography (SEC)

TMGMV samples (500 μL at 0.5 mg/mL) were analyzed using a Superose6 Increase 100 GL column and an ÄKTA Pure25 chromatography system (GE Healthcare, Chicago, Il, USA) using a flow rate 0.5 mL/min in 10 mM KP (pH 7.4). The absorbance at 260 and 280 nm was recorded.

### Circular dichroism spectroscopy (CD)

CD spectra were obtained using an Aviv model 215 CD spectrometer (Lakewood, NJ, USA). All samples were run in a quartz cuvette with a path length of 2 mm (Starna Cells, Atascadero, CA, USA) at 25 °C. Samples were dissolved in a 10 mM KP buffer at pH 7 to a concentration ranging from 0.025 mg/mL to 0.5 mg/mL to obtain a volume of 400 μL for each CD run. Near and far UV spectra were obtained in separate scans. For far UV spectra, samples were scanned from 250 to 180 nm with a wavelength step of 1 nm and an averaging time of 1 s. For near UV spectra, samples were scanned from 310 to 240 nm with a wavelength step size of 0.5 nm and an averaging time of 1 s. All spectra were scanned twice and averaged within each UV region.

### Small molecules quantification by high-performance liquid chromatography (HPLC)

For HPLC, AI was extracted from TMGMV. In brief, the concentration of TMGMV samples was adjusted to 1.2 mg mL^−1^ in KP. The solution was diluted fourfold into a 1:1 acetonitrile/:methanol mixture and vortexed for 30 s. The solution was centrifuged at 10,000 × g for 10 min at 4 °C and the organic phase (bottom fraction) was collected and transferred to an HPLC 2 mL glass screw top vial (SureSTART, Thermo Scientific, Waltham, MA, USA).

After a tenfold dilution in acetonitrile, the extracted samples were injected at 500 µL and run on 5 µm C18 column (20 × 100 mm) using a Shimadzu LC-40 HPLC system (Columbia, MD, USA). The method was run at 0.5 mL min^−1^ in a gradient of acetonitrile and 0.02% (v/v) phosphoric acid for 15 min per sample. A photodiode array was used to collect absorbance values at 280 nm (FLP), 269 nm (CTD), 225 nm (IVM), and 330 nm (RIF). The absorbance values were fitted to a standard curve to identify sample concentration with N = 3.

### Soil mobility

Cylindrical tubes (50 mL) open at both ends were wrapped at each end with two layers of 2.5″ cheesecloth and secured on a laboratory clamp. Soil was prewetted and packed into the column. For soil mobility studies, TMGMV and infused TMGMV were applied to the top of the soil columns in a single aliquot, and then the column was flushed at 5 mL min^−1^ with DI water. Eluent was collected from the bottom in several fractions and centrifuged for 15 s. The supernatant was isolated and refrigerated at 4 °C until characterization by SDS-PAGE. Measurements were made in triplicates (N = 3).

### Generation of AI structures

The structures of the four AI molecules were generated in ChemDraw 20.0 (PerkinElmer) and passed to Chem3D 20.0 (PerkinElmer). The structures were energy minimized using the MM2 function in Chem3D, and the surface mesh was generated by the software using the Total Charge Density (Huckel Calculation) feature.

### Docking and energy analysis

AutoDock Tools 1.5.6 (Scripps Research) and Chem3D 20.0 (PerkinElmer) were used for molecular docking simulations according to the guidelines given by AutoDock. In brief, TMGMV coat protein (PDB: 1vtm) was loaded and selected as the receptor molecule. The previously generated energy minimized structures of the AIs were selected as ligands, one AI per simulation. A coarse model of 1 Å grid spacing was generated to span the entire coat protein (70 X points, 60 Y points, 60 Z points). The default energy field generated by AutoDock Tools was selected for 50 individuals, 27,000 generations, 250,000 energy evaluations, and 20 docking poses per simulation. The 20 conformations were analyzed to extract stabilizing residues, relative position on the TMGMV coat protein, and heats of binding for each conformation.

## Results and discussion

We investigated the assembly/disassembly phase diagram of TMGMV and determined conditions amenable to AI entrapment. The primary goal was to achieve “breathing” without full disassembly. First, we focused on pH-induced structural changes and AI infusion. In this approach the process required extensive parameter optimization, including pH (7 to 8), incubation time (2 to 24 h), protein concentration (100 to 500 AI equivalences per CP), as well as the AI addition intervals (one feed vs. daily increments). The latter was a critical parameter: bulk additions led to severe aggregation and insolubility—likely as a result from the hydrophobic AI binding to the nanoparticle surface promoting interparticle association and aggregation. We determined best results were obtained when the AI was added in daily increments over 10 days; this assured particle stability and AI loading (see below). In brief, 10 equivalences of AI per CP were fed daily at pH 7.5 and then left stirring overnight; this process was repeated for 10 days. After completion, excess reagents were removed by spin filtration, samples reconstituted in buffer, and stored at 4 °C until further processing.

DMSO was used to disrupt inter-coat protein interactions as a second approach. For some AI, in particular those that are highly hydrophobic (e.g. IVM and FLP), this was favorable. The benefits of this approach are two-fold: increased solubility of AI leads to a higher effective concentration to drive infusion and the cosolvent prevents AI precipitation which interferes with the infusion process. To further improve on this process, the TMGMV preparations were subjected to magnetic stirring and fed from the top of the tube, preventing any short-term spikes in AI concentration that may promote precipitation. For ivermectin and fluopyram, both compounds exhibited immediate precipitation under the ‘pH approach’. In stark contrast, the ‘DMSO approach’ demonstrated to be feasible with no visible aggregates. Additionally, the increased agitation (continuous stirring), bond disrupting effect of DMSO, and higher concentrations of AI in solution—all factors should drive AI infusion more quickly helping to maintain the TMGMV nanoparticle structure avoiding aggregation. The optimized procedure is shown in Figure S1 and described in methods.

**Figure 2:**
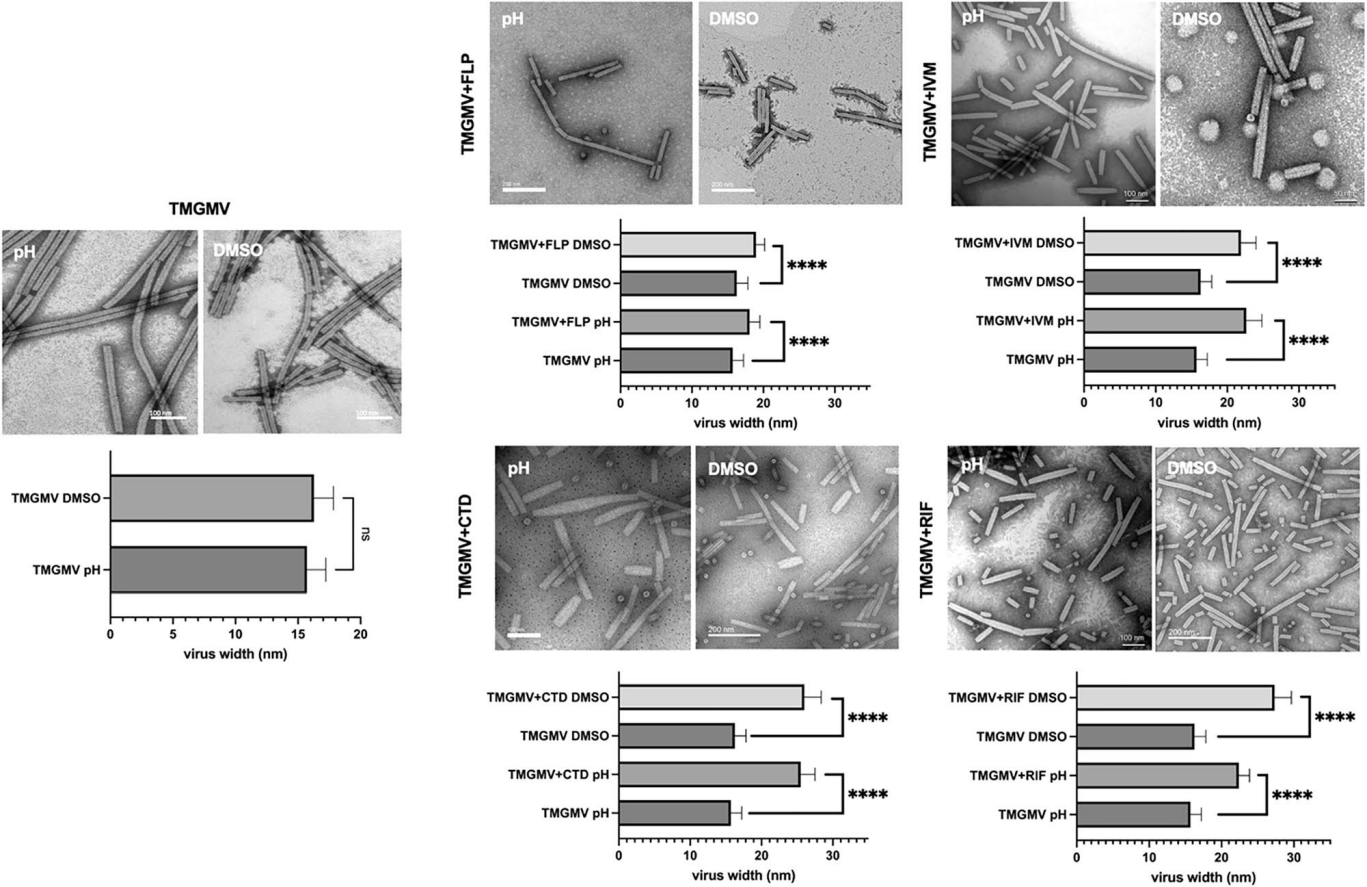
TEM and image analysis of the non-covalently conjugated viral nanoparticles. ****p-value is < .00001, n = 30. CTD = clothianidin, FLP = fluopyram, IVM = ivermectin, RIF = rifampicin.

TEM imaging was performed and rod shaped virions were observed (Fig. 2). Most intriguing was the notable structural change upon AI-loading: AI-laden TMGMV appeared swollen and the structural transitions suggested AI entrapment. To gain insights into the degree of structural changes, quantitative TEM image analysis was performed comparing native vs. the AI-laden TMGMV (n = 30). Based on the negative stain, native virions were 15.7 nm in width in average (± 1.9 nm), which is an underestimation to native TMGMV of 18 nm. This could be due to the uranyl acetate negative staining yielding a heavy shadow on the borders of the virion; this artifact may lead to the apparent reduced width. While there was no statistical significance between native TMGMV and controls that underwent both “breathing” methodologies with no AI added, for each AI added the increment in comparison to native TMGMV was observed in Fig. 2. FLP- and IVM-loaded TMGMV displayed a maximum width of 18 or 23 nm, respectively. In other words, fluopyram loading resulted in a 14% width increase and ivermectin-loading resulted in a 46% width increase compared to native TMGMV (*p*-value =  < 0.00001). Meanwhile, CTD and RIF loading produced 22–27 nm rods, significantly thicker than native TMGMV; the width increase for clothianidin and rifampicin was 65% and 73%, respectively, compared to native TMGMV (*p*-value =  < 0.00001).

**Figure 3:**
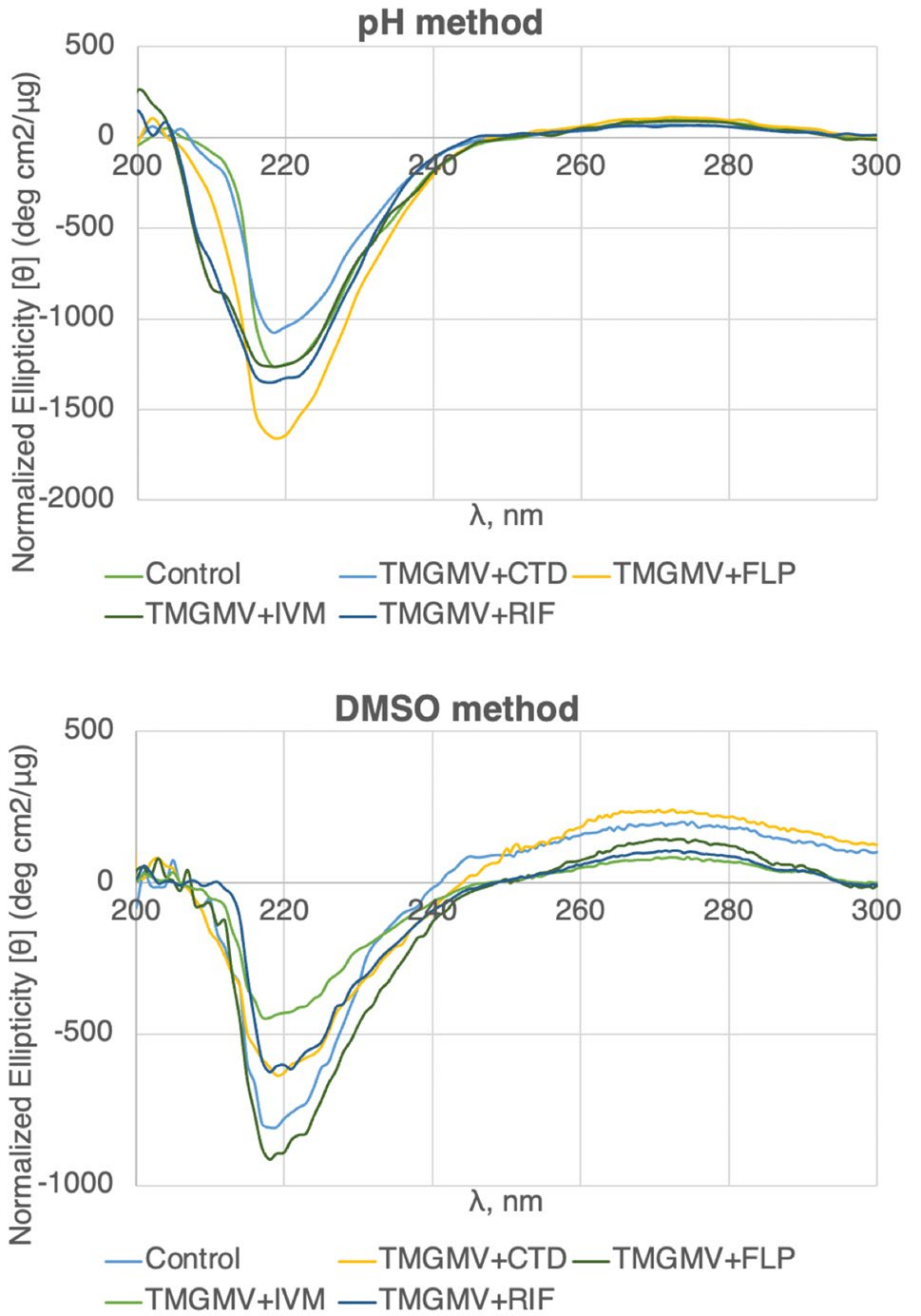
Circular dichroism spectra for TMGMV samples loaded with AI as indicated (control = native TMGMV). CTD = clothianidin, FLP = fluopyram, IVM = ivermectin, RIF = rifampicin.

Next, to observe any possible alterations in the secondary structure of TMGMV after exposing the particles to breathing and infusion, CD was performed (Fig. 3). The effects of structural motifs on CD are additive and can be challenging to deconvolute^46^. Rather, the differences between spectra of treatment groups can signify whether or not structural changes occurred. The most intense signal for protein or virus CD is around 205–220 nm, which represents the sum of contributions from alpha helices, beta sheets, and random coil. The shift of the global minimum from 208 to 220 nm suggests a larger contribution from aggregation behavior or alpha helical content than beta sheets in both, the pH and DMSO samples. Other than that, CD indicated no changes in structure, and this is as expected. In this protocol, CPs are dissociated yet not denatured—thus, there is no expectation for the CP structure itself to change. Rather the supramolecular assembly is altered with inter-coat protein interactions likely distorted through the AI cargo. In the range of 208–220 nm, the AIs tested all showed similar molar ellipticity profiles, suggesting there were no differences in the secondary structure. In the near UV range (250–320 nm), where RNA is the highest contributor to these spectra, a similar trend holds with the AIs, showing each shares a general shape of signal profile. These results suggest that both the pH and DMSO approaches for breathing do not significantly alter the secondary structure of TMGMV. In summary, the modest pH elevation and relatively low volume fraction of DMSO used for these breathing experiments were not expected to alter the secondary structure of the virion or its CP, but rather the tertiary structure of the nucleoprotein assembly to allow inter-coat protein loading^47,48^.

To further verify structural integrity of the AI-laden TMGMV particles post processing and purification, SEC was performed. SEC measurements showed no significant difference between native and AI-laden TMGMV for any AI showing the typical elution profile from the Superose6 Increase column with elution at  ~ 10 mL and an A_260:280_ ratio of 1.2, indicative of intact TMGMV, where 260 nm denotes RNA absorption and 280 nm protein absorption (Figure S2). However, it is of note that SEC does not provide the resolution to show differences in width or length of TMGMV nanoparticle formulations.

**Figure 4:**
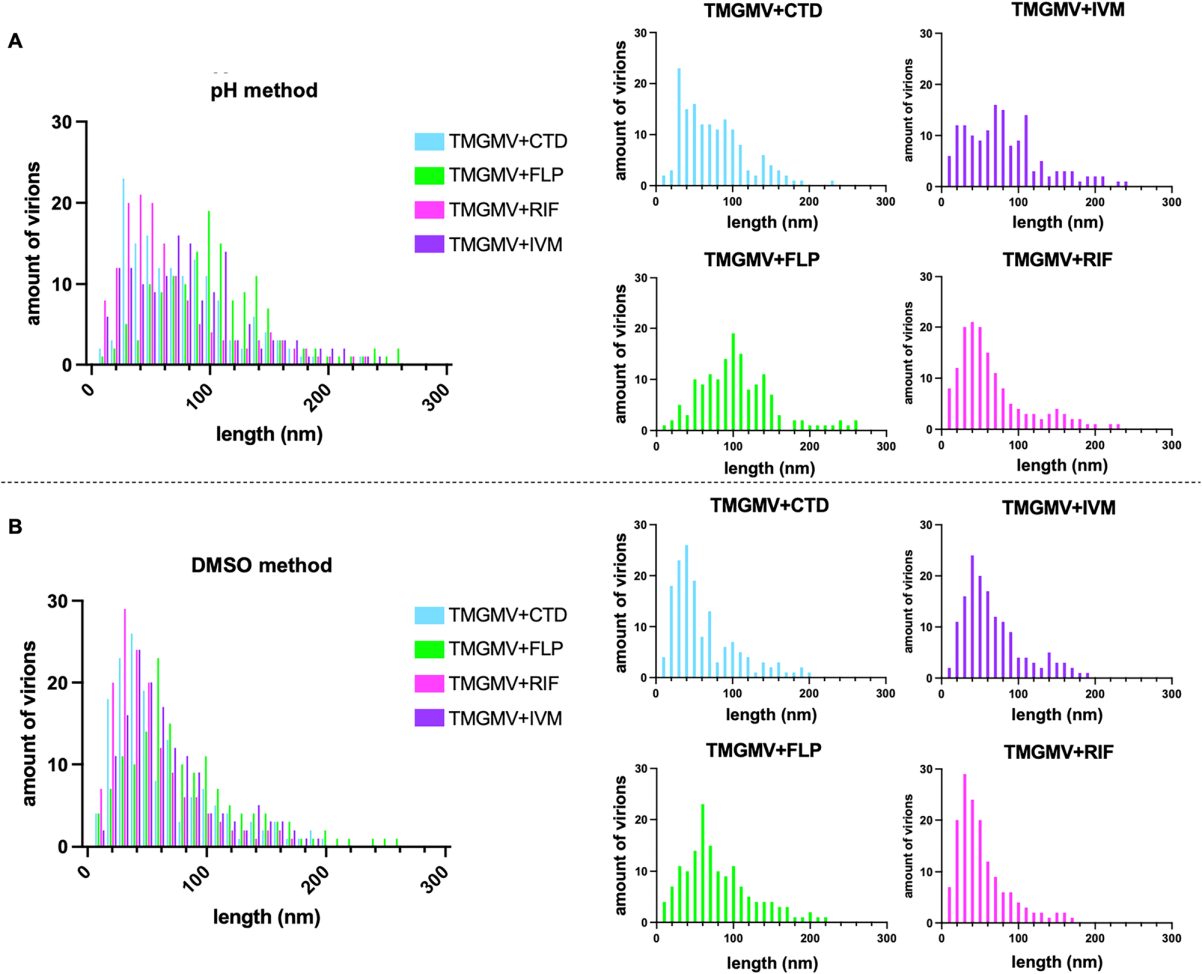
Comparison of the length of virions after AI infusion. Image analysis from transmission electron microscopy: (**A**) pH breathing methodology; (**B**) DMSO-induced breathing methodology. CTD = clothianidin, FLP = fluopyram, IVM = ivermectin, RIF = rifampicin.

In addition to changes in the width of the AI-laden TMGMV, we noted differences in the length of the nucleoprotein assemblies with or without AI. Overall TMGMV samples are heterogeneous in length; several disks were apparent indicating partial disassembly and breakage. Also, blob-like structures were observed in the IVM sample. These are believed to be IVM aggregates of precipitated of the highly hydrophobic IVM (Fig. 2). Nanoparticle length was measured through image analysis using ImageJ software. Overall, AI-laden samples were shorter compared to treated (but not AI-laden) or untreated TMGMV: the pH treatment showed slightly less breakage, having a higher distribution of lengths, compared to the DMSO treatment (Fig. 4). DMSO treatment showed the majority of the TMGMV below 100 nm, and this was independent of AI or loading method.

Using HPLC, we proceeded to quantify the TMGMV-loaded AI (Table 1). Using the pH-based method for infusion, CTD and RIF showed successful loading after 10 days of batch loading the AI in solution, achieving  ~ 1108 molecules per virion for clothianidin and 738 molecules per virion for rifampicin. In the cases of fluopyram and ivermectin, a large amount of precipitation was observed when the pH method was used for AI loading; this likely stripped the virus from solution and made the AI inaccessible for diffusion into the virus. Therefore, there was negligible loading of FLP or IVM per TMGMV (see Table 1). When comparing these results to the TEM micrographs and changes in the aspect ratio of the AI-laden TMGMV, the amount of AI per virion and changes in morphology appear to correlate: the more AI is loaded the larger the width increase. CTD and RIF resulted in the significant width increases (> 65% over native TMGMV), while FLP only resulted in  < 15% width increase. IVM may be an outlier: while IVM-loading was not apparent, significant changes in morphology were observed and this may be attributed to challenges in extraction of IVM or molecular properties of IVM that permanently distort the structure of TMGMV without permanent loading of the AI.

**Table 1:** Quantification of AI in samples by HPLC using the pH and DMSO infusion techniques.

	Fluopyram		Clothianidin		Ivermectin		Rifampicin	
	pH	DMSO	pH	DMSO	pH	DMSO	pH	DMSO
Detected in sample	0.05 µg/mL	0.61 µg/mL	2.29 µg/mL	2.06 µg/mL	0.02 µg/mL	0.44 µg/mL	4.9 µg/mL	7.52 µg/mL
Molecules per virion	15.82	185.59	1107.55	995	2.89	61.63	737.66	1104.2
Molecules per CP	0.007	0.087	0.52	0.47	0.001	0.029	0.35	0.51

Using the DMSO method, the AI loading efficiency for FLP and IVM was improved. FLP loading resulted in 186 moieties per virion, which is a  ~ 12-fold increase compared to the pH method. A similar result was observed for IVM, with a  ~ 21-fold increase allowing  > 60 molecules to be loaded per virion. However, for agriculture applications, the IVM loading is considered low and would require further optimization (for example, we recently demonstrated an alternate and efficient loading strategy for IVM by use of spherical protein nanoparticles^49^). Interestingly for CTD, the DMSO method was slightly less effective with 995 moieties per virion loaded (a 10% decrease over the pH method), and RIF-loading has a modest increase aching 1.5-fold higher loading reaching 1104 molecules per virion.

Lastly, to probe robustness of the methods, we also performed loading of a chemotherapeutic (DOX) and near-infrared fluorophore (Cy5) with utility in nanomedicine (drug delivery and imaging). In each case, swelling of the TMGMV diameter was observed by TEM while SEC confirmed intactness of the assembly and co-localization of the fluorescent cargo with the TMGMV carrier. Both DOX and Cy5 are fluorescent molecules, there loading was quantified using absorbance spectra and their extinction coefficients resulting in  ~ 615 DOX per TMGMV and  ~ 80 Cy5 per TMGMV (Figure S3).

Together, we developed a pH and solvent-based approach to load various AIs into TMGMV nanorods. AI-infused TMGMV particles remain intact yet are swollen as a function of the AI. Data indicates that for some compounds the DMSO method may be favored. There is room for further optimization and tailoring the infusion period, cosolvent choice and concentration, mixing rates, and increments of AI as well as its concentration added.

Soil mobility studies indicated that there is a similar distribution of the non-treated and treated (non-loaded) TMGMV throughout the soil (Figure S4, original blots/gels are presented in Figure S5). These suggest that there is no alteration of the properties of the virus and its ability to move through soil. Assays performed with treated and loaded (DOX or Cy5) TMGMV, it could be observed that the cargo remained with the virus and got distributed through the soil column similarly as non-loaded TMGMV (Figure S4, original blots/gels are presented in Figure S5). These indicates that the creation of these pockets favors the interaction of the hydrophobic cargo and the virus particle, allowing it to travel further into the soil, instead of it being very exposed to the surface and having it interact strongly with the soil.

**Figure 5:**
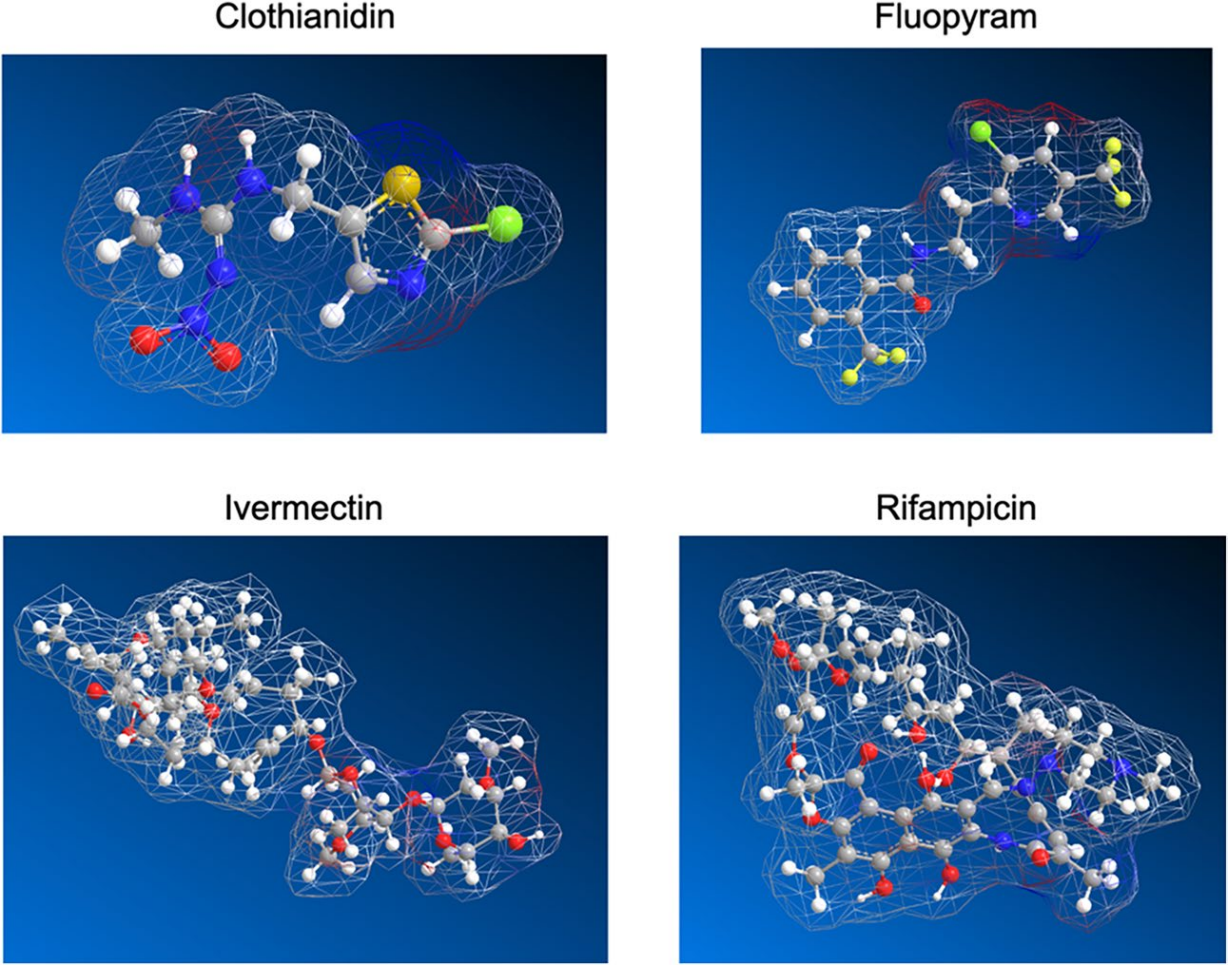
Surface charge distribution of AI molecules. A ball and stick model of the Ais with a space-filling mesh surface surrounding them. Atoms are colored so grey is carbon, white is hydrogen, blue is nitrogen, red is oxygen, yellow is sulfur, chlorine is green, and chartreuse is fluorine. The surface mesh is colorless if there is no electrostatic potential, blue if positive, and red if negative. The conformations of the molecules are energy minimized.

To gain a better understanding of the molecular properties that lead to inter-coat protein loading of Ais, we compared their aqueous and organic partition coefficients (logP), molecular weights, and surface charge distributions. A summary of these properties can be found in Table 2 and Fig. 5. IVM and FLP had the highest logP values of 4.4 and 3.33, respectively. CTD and RIF had values of 1.3 and 2.4, respectively^50–52^. The values for IVM and FLP indicate the molecules are water insoluble, which matches well to our observations in the loading experiments. This could explain why the same effective morphology changes using these Ais was achieved within 1 day using DMSO versus 10 days for the pH approach, as the effective concentration of AI in solution was much higher. Another factor to consider regarding infusion efficiency is their size. It is possible that larger molecules could have steric hindrance when entering the spaces between coat proteins during these measurements. When we analyzed the changes in particle width using both approaches compared to their molecular weights, we see clothianidin (249.68 Da) had the largest change in width and FLP (396.71 Da) had the smallest change in width. RIF (822.94 Da) and IVM (875.1 Da) had intermediate values for changes in width. There is no clear trend in this set based on molecular weight, so AI size does not seem to be the limiting factor for loading using this approach.

**Table 2:** Comparison of the Ais aqueous and organic partition coefficients (logP) and molecular weights.

Molecule	MW (Da)	logP
Cyanine 5	653.78	3.84
Doxorubicin	543.52	0.02
Fluopyram	396.71	3.33
Clothianidin	249.68	1.3
Ivermectin	875.1	4.4
Rifampicin	822.94	2.4

Beyond the range of small molecules, steric hindrance would be expected to dominate. From the electron density plots of the Ais, we observed ivermectin and rifampicin have large regions with no charge and small regions of small charge that are largely separated, creating mildly amphiphilic molecules. On the contrary, FLP and CTD are much smaller and have a higher surface area of charge. Because TMGMV is zwitterionic in nature but also contains many hydrophobic interfaces, it is challenging to isolate the predicted changes in morphology to a single physicochemical interaction. The amphiphilic, charged, compact, and flexible structure of clothianidin may all work together to alter the morphology of TMGMV.

**Figure 6:**
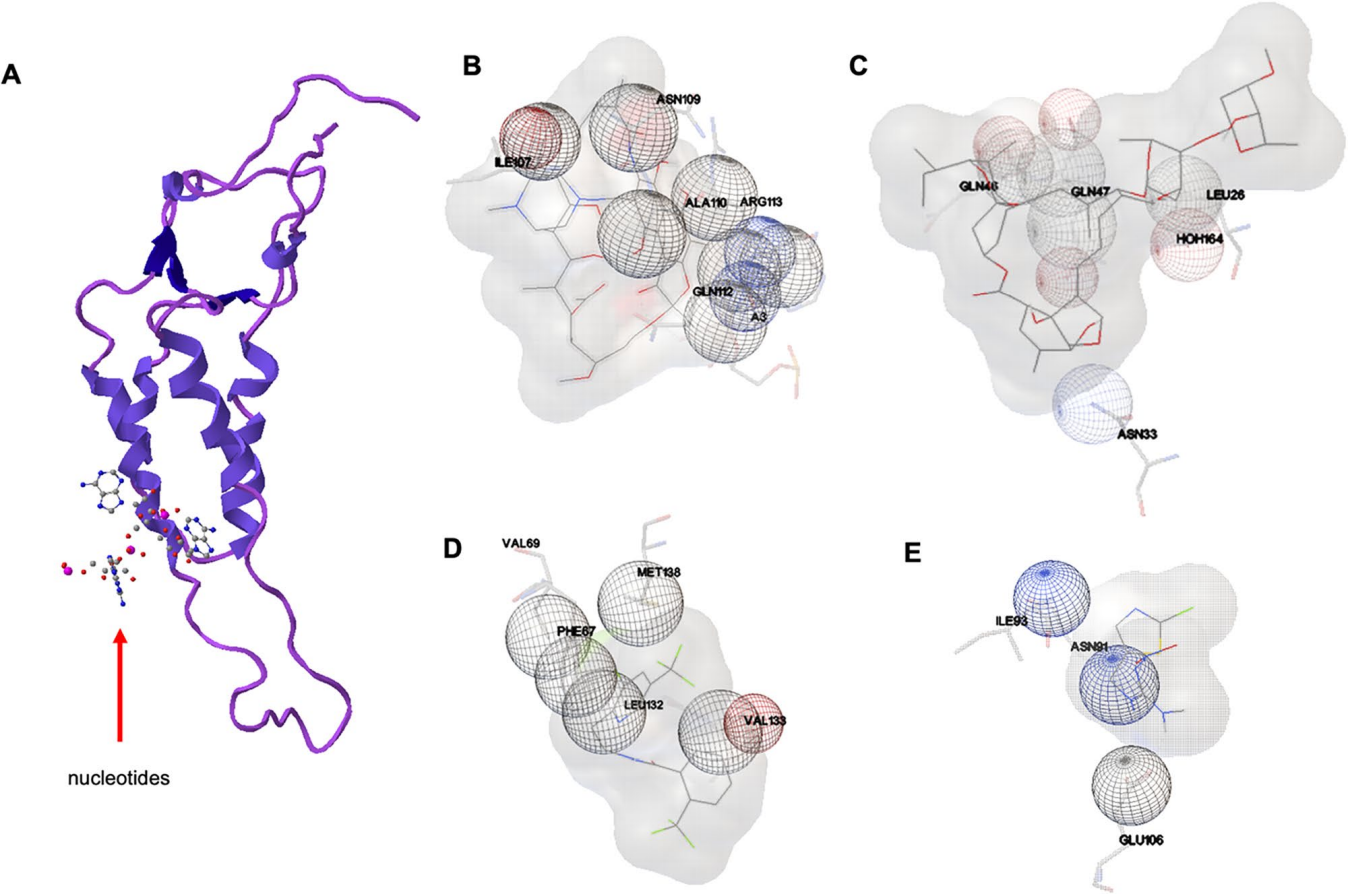
Docking of AI to TMGMV. TMGMV coat protein (PDB: 1VTM) where red arrows indicate the location of nucleotides (**A**). A space filling model of a putative binding site for RIF (**B**), IVM (**C**), FLP (**D**), and CTD (**E)**. Red and blue spheres indicate negative and positive electrostatic contributions, respectively. The three-letter amino acid abbreviation are followed by the residue number.

To gain some insight into how the Ais interact with the coat protein surface, molecular docking experiments of TMGMV CP (PDB: 1VTM) and the four Ais were conducted. In the analysis, the top 20 docking conformations were analyzed for their binding energy and the residues involved in stabilizing the AI. These data do not suggest the Ais are proper ligands for TMGMV CP, but rather identify putative residues that may be implicated in inter-coat protein loading. True ligand interactions have been reported for heats of binding greater than 8 kcal mol^−1^, while a majority of these interactions fall within 3–8 kcal mol^−1^^53,54^. Table S1 summarizes the regions of binding, their function for TMGMV, and the residues specifically identified to stabilize the AIs. Figure 6 shows examples of docked AIs on TMGMV and the implicated residues, and Figure S6 shows the heats of binding for each conformation as calculated by Autodock 4. From the simulated docking, we observe that of the 20 best binding sites on TMGMV CP, all 4 AIs have many sites that are likely inaccessible. Depending on the mechanism of separation of TMGMV CPs (between CPs versus between disks), there are up to 10 accessible sites for IVM, 8 for RIF, 11 for FLP, and 5 for CTD. The binding energy distribution in Figure S6 shows rifampicin has the highest heats of binding to the surface, followed by ivermectin, then FLP and CTD. Despite a high number of potential binding sites, IVM is a very large molecule and would require a high degree of separation of CPs to intercalate into the virion. Its relatively high affinity may manifest in transient surface binding which can disrupt inter-coat protein bonds, explaining the widening of TMGMV in the presence of IVM. Ultimately, the IVM is not detectable during quantification, suggesting it does not stay bound to TMGMV. RIF has the highest heat of binding to TMGMV CP, loads well onto TMGMV, and induces morphological changes on TMGMV. It shows improved loading in the presence of DMSO compared to the pH approach, suggesting the structural changes induced by DMSO allow this relatively large molecule access to binding sites. Despite having 11 potential binding sites, FLP also had some of the lowest heats of binding and had the highest affinities for the inner channel. Because this molecule is insoluble and relatively small, it may preferentially partition to the inner channel than to load between the CPs. CTD had 5 accessible sites on the exterior according to the docking model, but also had some of the lowest binding energies. However, its relatively small size and surface charge distribution may have aided in its binding and disruption of structure between TMGMV CPs. CTD demonstrated some of the highest loading by HPLC and largest differences in virion width, suggesting the properties of this molecule make it well suited for this approach. With more robust docking analysis and a larger library of small molecules to load between TMGMV CPs, it may be possible to pinpoint molecular properties of the AIs and individual residues of TMGMV CP that are implicated in these binding events.

## Conclusions

We developed strategies that allow functionalization of TMGMV by inter-coat protein loading with agriculturally relevant cargos, including fluopyram, clothianidin, rifampicin and ivermectin. This was achieved by careful adjustment of the bathing conditions, priming structural changes and “breathing” of nucleoprotein assemblies facilitating integration of hydrophobic cargos. We also demonstrated that these methods are robust and could be extended for functionalization with therapeutic molecules or imaging moieties used in nanomedicine applications. Intriguingly the AI-laden TMGMV appear swollen and the increase in width is correlated with AI cargo loading. Therefore, the change in TMGMV width may serve as a preliminary assessment to track the degree of loading. This data highlights that virus-based nanostructure are dynamic and modular systems. Through adjustment of the bathing conditions, inter-coat protein interactions are weakened allowing cargos to be trapped in molecular inter-coat protein pockets—likely through a combination of hydrophobic, van der Waals and electrostatic interactions. While for some cargo pH-mediated loading was efficient, for other cargo co-solvents approaches were most suitable. This indicates that there is room for optimization and cargo-tailored methodologies to be developed. Structural modeling as employed here will help guide the design and to device structure-based engineering approaches toward development of rules for efficient AI-loading into TMGMV and other high aspect ratio soft matter nanoparticle systems. Future work aims to further explore and optimize the release kinetics of active ingredients encapsulated within the TMGMV system, focusing on enhancing controlled and targeted release mechanisms for more effective disease and pest control in agricultural applications.

### Supplementary Information

Supplementary Information.

## Data Availability

The data that support the findings of this study are available on request from the corresponding author, NFS.
