# Supplementary material for: Inter-coat protein loading of active ingredients into Tobacco mild green mosaic virus through partial dissociation and reassembly of the virion
Source: Sci Rep. 2024 Mar 26;14:7168. doi: 10.1038/s41598-024-57200-0 (PMC10965923; doi:10.1038/s41598-024-57200-0)
Supplement: Supplementary file 1 — Supplementary Information. [file 41598_2024_57200_MOESM1_ESM.docx]

**Supporting Information**

**Inter-coat protein loading of active ingredients into *Tobacco mild green mosaic virus* through partial dissociation and reassembly of the virion**

Ivonne González-Gamboa^[a][d][h][j]^, Adam A. Caparco^[a][h]^, Justin McCaskill^[a]^, Paulina Fuenlabrada-Velázquez^[a]^, Samuel S. Hays^[a]^, Zhicheng Jin^[a]^, Jesse V. Jokerst^[a][i]^, Jonathan K. Pokorski^[a][d][e]^, Nicole F. Steinmetz^[a]-[h]^*

[a] Department of NanoEngineering, University of California, San Diego, La Jolla, CA, USA

[b] Department of Bioengineering, University of California, San Diego, La Jolla, CA, USA

[c] Department of Radiology, University of California, San Diego, La Jolla, CA, USA

[d] Center for Nano-ImmunoEngineering, University of California, San Diego, La Jolla, CA, USA

[e] Institute for Materials Discovery and Design, University of California, San Diego, La Jolla, CA, USA

[f] Moores Cancer Center, University of California, San Diego, La Jolla, CA, USA

[g] Center for Engineering in Cancer, Institute of Engineering in Medicine, University of California, San Diego, La Jolla, CA, USA

[h] Shu and K.C. Chien and Peter Farrell Collaboratory, University of California, San Diego, La Jolla, CA, USA

[i] Materials Science and Engineering Program, University of California San Diego, 9500 Gilman Dr, La Jolla, CA, USA

[j] Department of Molecular Biology, University of California, San Diego, La Jolla, CA, USA

*corresponding author: nsteinmetz@ucsd.edu

**Supplemental Figures and Tables**


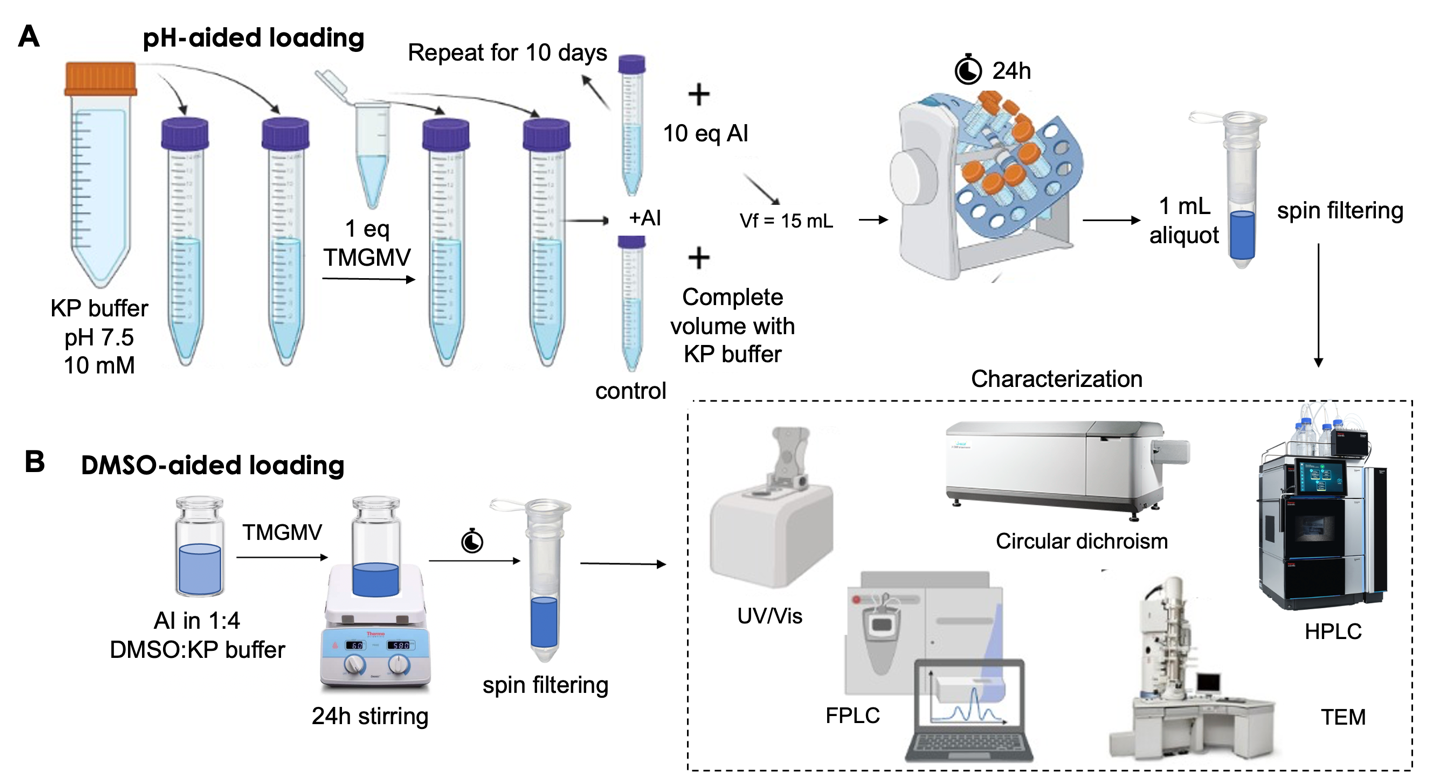


**Figure S1.** Schematic representation of the methodology for infusion of AI via the pH method (A) and DMSO method (B).


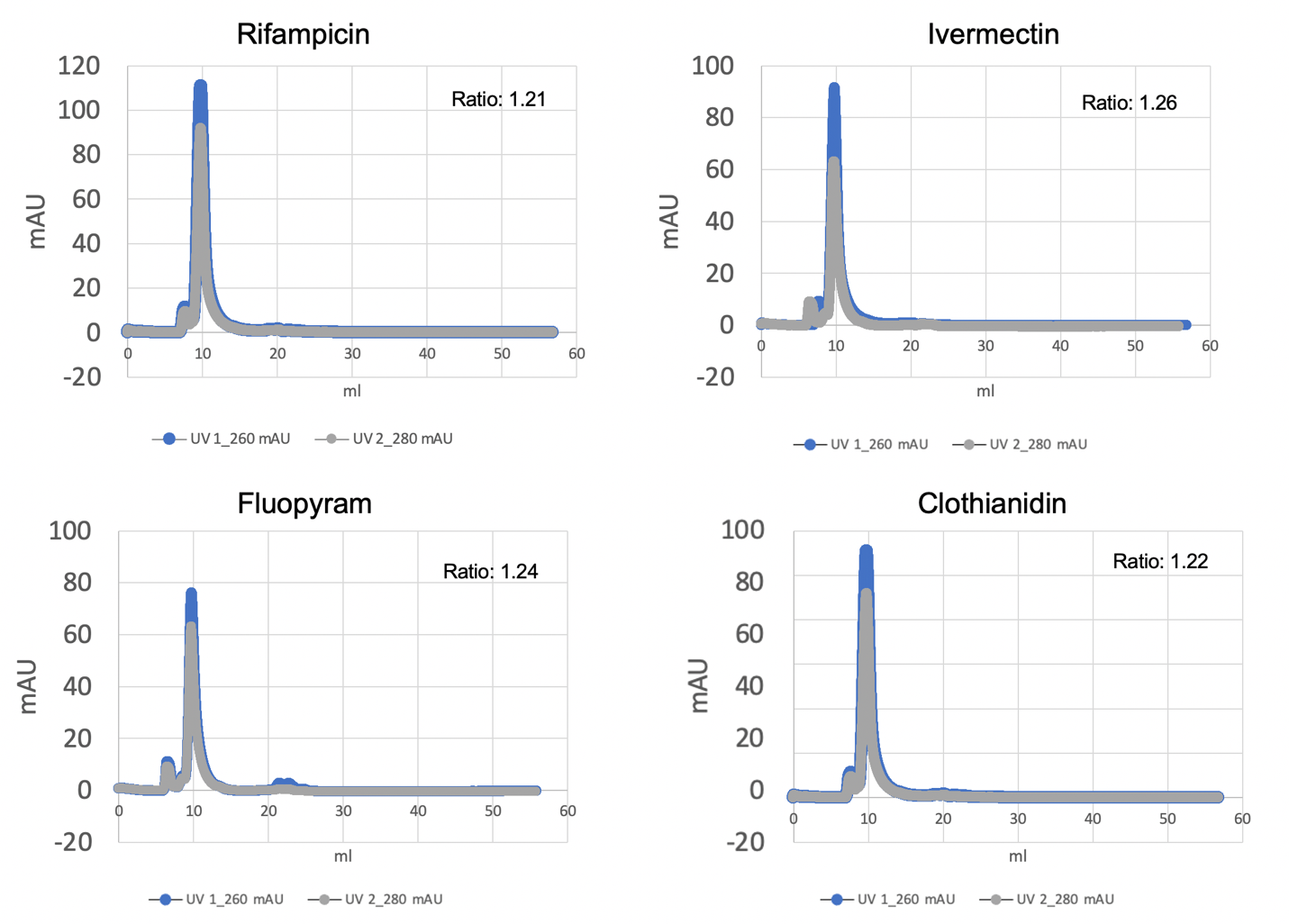


**Figure S2.** Size exclusion chromatography of AI-loaded TMGMV. A_260:280_ ratio, where 260 nm denotes RNA absorption and 280 nm protein absorption.


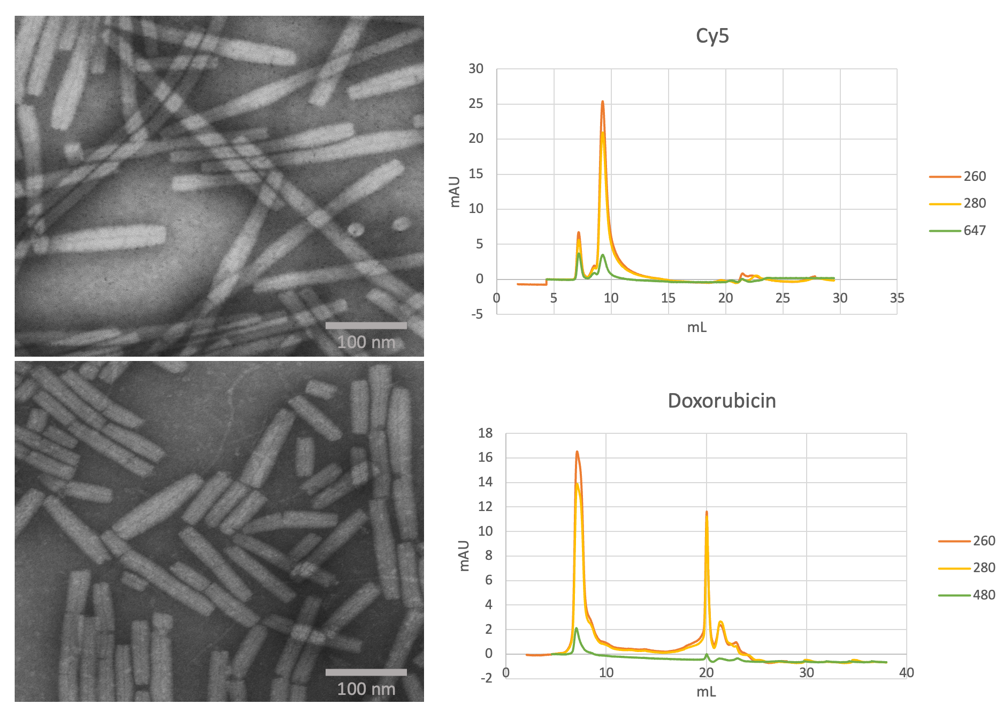


**Figure S3.** Cy5 and DOX-loaded TMGMV: transmission electron microscopy (left) and size exclusion chromatography (right).


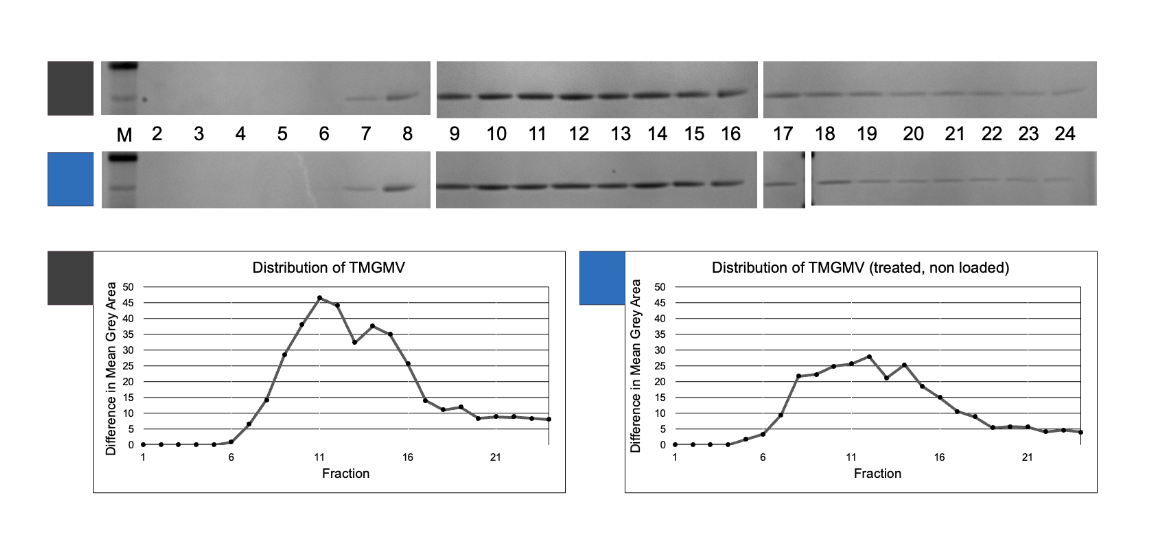


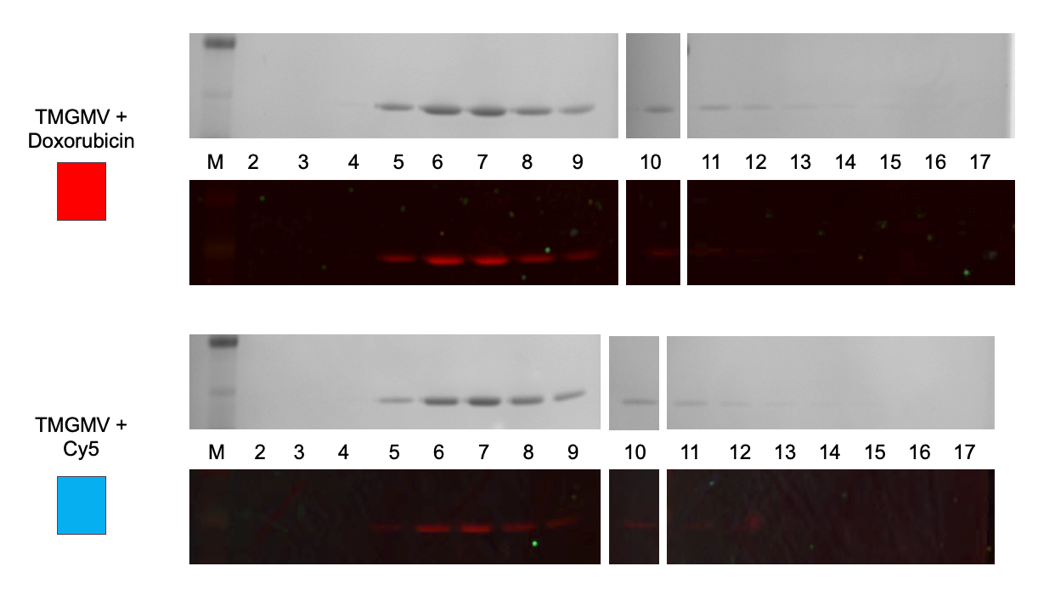


**Figure S4.** Soil mobility assays of non-treated TMGMV (black square) and treated (pH method), but non-loaded TMGMV (blue square), TMGMV + Doxorubicin (pH method, red square) and TMGMV + Cy5 (pH method, light blue square). Fractions were collected and loaded into a SDS gel. Cropped images for clarity. Uncropped version can be observed in Figure S5.

**
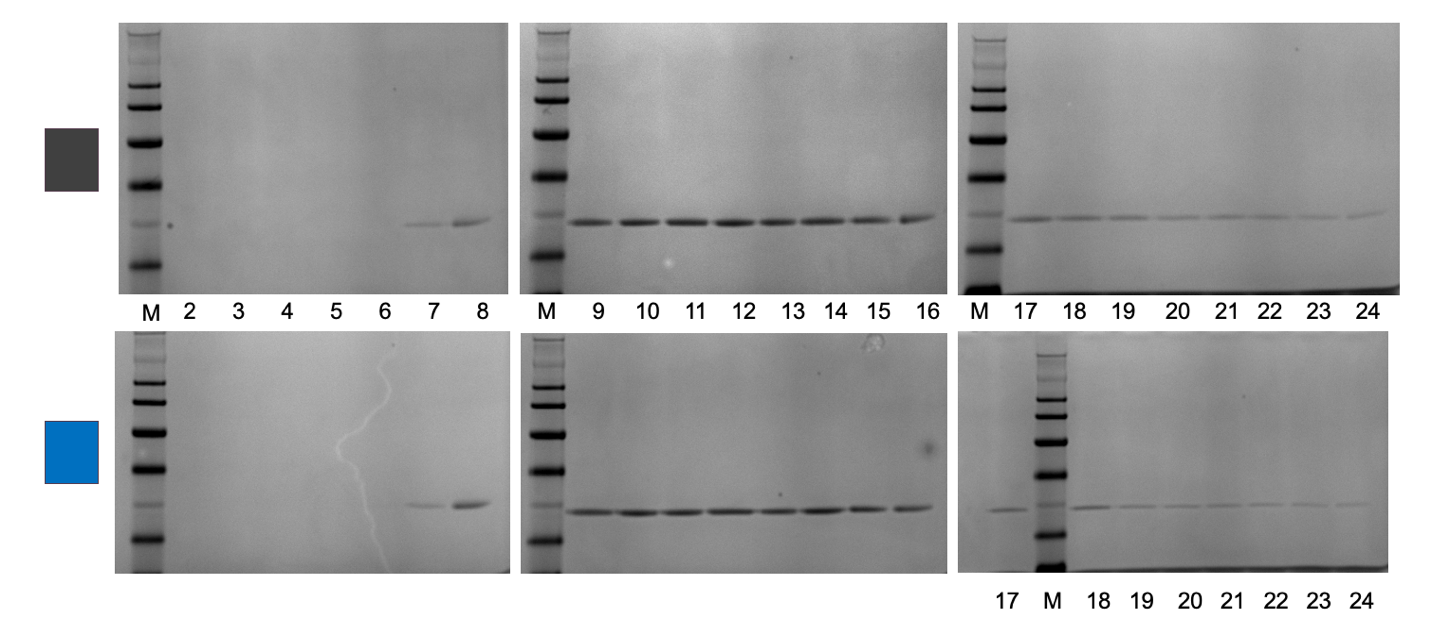

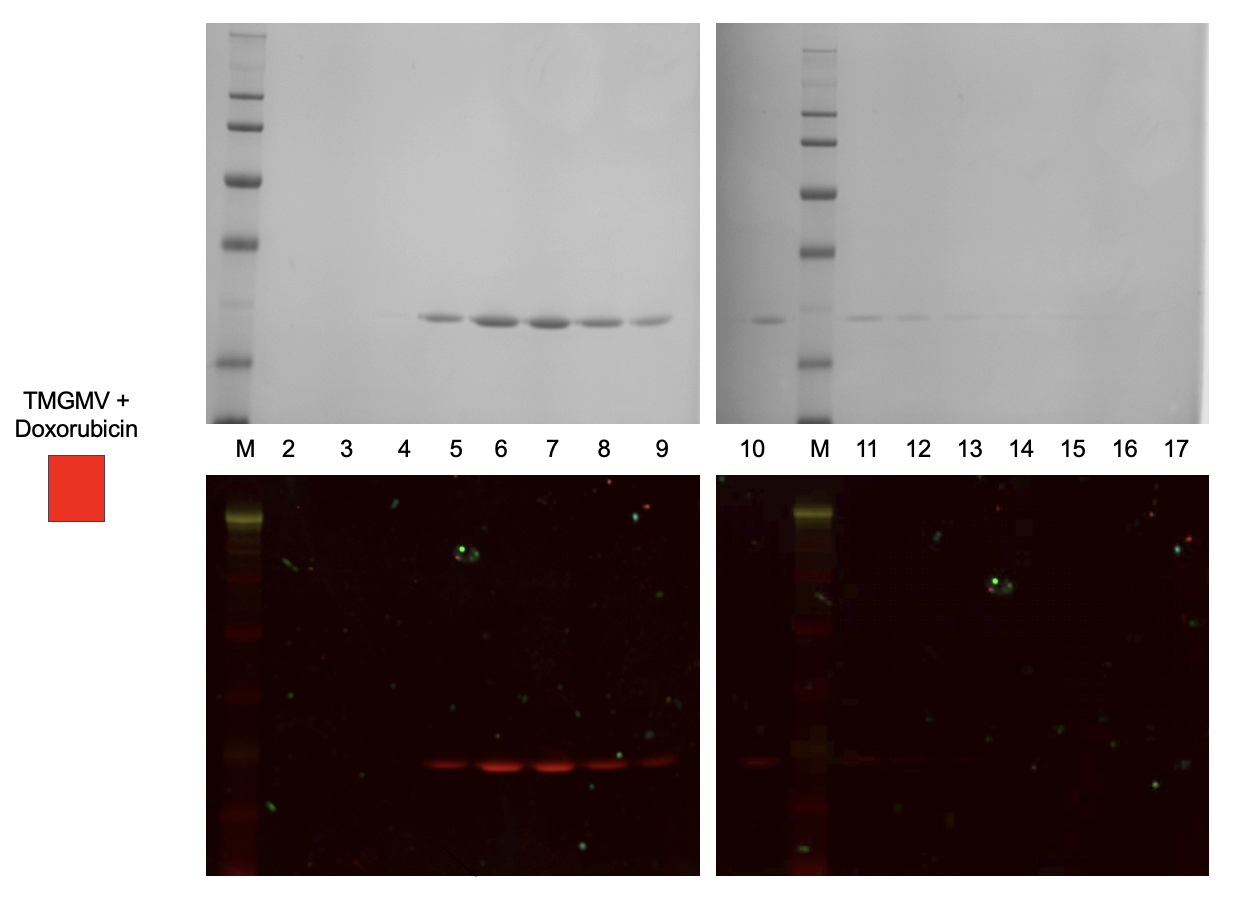
**

**
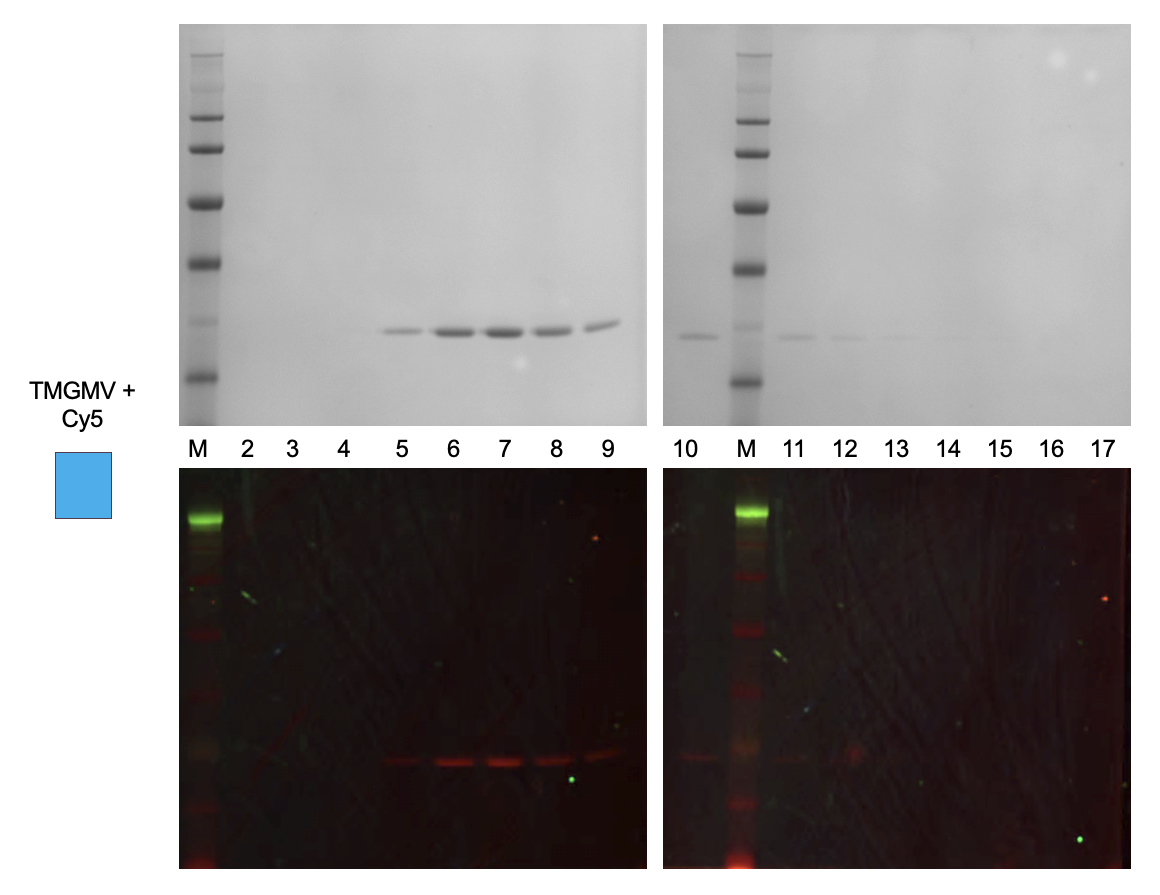
**

**Figure S5.** Original blots/gels presented on Figure S4.

**Table S1.** Regions of binding, their function for TMGMV, and the residues specifically identified to stabilize the AIs. The top 20 results for docking simulations of AIs and an individual TMGMV coat protein (PDB: 1VTM). The AIs are fluopyram, clothianidin, ivermectin and rifampicin,. The results are organized by decreasing heats of binding from the simulation. The location of interaction, likelihood of accessibility, and implicated residues are given for each conformation.

| **Fluopyram** | | | | | | | | | | | | | |  |  |  |
| --- | --- | --- | --- | --- | --- | --- | --- | --- | --- | --- | --- | --- | --- | --- | --- | --- |
| **Docking #** | | **Region** | **Residues** | | | | | | | | | | |  |  |  |
| 1 | | inner channel | T111 | | | N91 | | I93 | I94 |  |  |  | |  |  |  |
| 2 | | inner channel | Q38 | | | R90 | | R92 | N91 | I93 |  |  | |  |  |  |
| 3 | | interior; likely inaccessible | R92 | | | I94 | | R90 | V114 |  |  |  | |  |  |  |
| 4 | | interior; likely inaccessible | Y72 | | | R71 | | W52 | L64 | F48 |  |  | |  |  |  |
| 5 | | interior; likely inaccessible | Q38 | | | R90 | | R92 | N91 | Q36 |  |  | |  |  |  |
| 6 | | interior; likely inaccessible | R71 | | | L84 | | A49 |  |  |  |  | |  |  |  |
| 7 | | exterior; likely accessible | M138 | | | V133 | | F67 | V69 | P20 | I24 | V21 | |  |  |  |
| 8 | | interior; likely inaccessible | S86 | | | T89 | | R90 |  |  |  |  | |  |  |  |
| 9 | | exterior; likely accessible | M138 | | | F67 | | V69 |  |  |  |  | |  |  |  |
| 10 | | exterior; likely accessible | M138 | | | F67 | | L132 |  |  |  |  | |  |  |  |
| 11 | | exterior; likely accessible | G135 | | | M138 | | G137 | F139 | G143 | T146 |  | |  |  |  |
| 12 | | exterior; likely accessible | T146 | | | G143 | | G137 | F139 | M138 |  |  | |  |  |  |
| 13 | | interior; likely inaccessible | A120 | | | A117 | | D116 | S86 | T89 |  |  | |  |  |  |
| 14 | | exterior; likely accessible | V133 | | | L132 | | M138 | F67 | V69 |  |  | |  |  |  |
| 15 | | exterior; likely accessible | V69 | | | F67 | | L132 | I24 | V21 | P20 |  | |  |  |  |
| 16 | | exterior; likely accessible | R61 | | | T59 | |  |  |  |  |  | |  |  |  |
| 17 | | inner channel | R92 | | | I93 | | V96 | I94 |  |  |  | |  |  |  |
| 18 | | exterior; likely accessible | F67 | | | V69 | | M138 | G135 |  |  |  | |  |  |  |
| 19 | | exterior; likely accessible | F67 | | | I24 | | M138 | V133 |  |  |  | |  |  |  |
| 20 | | exterior; likely accessible | M138 | | | G137 | | F139 | A147 | T146 |  |  | |  |  |  |
| **Clothianidin** | | | | | | | | | | | | | |  |  |  |
| **Docking #** | | **Region** | **Residues** | | | | | | | | | | |  |  |  |
| 1 | | inner channel | I93 | | | N91 | | E106 |  |  |  |  | |  |  |  |
| 2 | | inner channel | N103 | | | I94 | | N91 | T111 | V114 |  |  | |  |  |  |
| 3 | | inner channel | I93 | | | N91 | | N103 | E106 |  |  |  | |  |  |  |
| 4 | | inner channel | I93 | | | I94 | | R92 | N91 | T111 | V114 |  | |  |  |  |
| 5 | | inner channel | I94 | | | N103 | | R90 | Q36 |  |  |  | |  |  |  |
| 6 | | interior; likely inaccessible | R113 | | | D116 | | A117 |  |  |  |  | |  |  |  |
| 7 | | interior; likely inaccessible | R92 | | | N91 | | R90 |  |  |  |  | |  |  |  |
| 8 | | interior; likely inaccessible | R92 | | | N91 | | R90 |  |  |  |  | |  |  |  |
| 9 | | interior; likely inaccessible | Q34 | | | T118 | | R122 | L31 |  |  |  | |  |  |  |
| 10 | | exterior; likely accessible | D19 | | | D66 | | Y68 | Y17 |  |  |  | |  |  |  |
| 11 | | interior; likely inaccessible | T118 | | | V119 | | R122 | Q34 | Q36 | D115 |  | |  |  |  |
| 12 | | exterior; likely accessible | W152 | | | E145 | |  |  |  |  |  | |  |  |  |
| 13 | | interior; likely inaccessible | A51 | | | K53 | | D19 | E22 |  |  |  | |  |  |  |
| 14 | | exterior; likely accessible | K53 | | | A51 | | E22 | L26 | Q47 |  |  | |  |  |  |
| 15 | | exterior; likely accessible | K53 | | | D19 | | E22 |  |  |  |  | |  |  |  |
| 16 | | interior; likely inaccessible | A117 | | | D116 | |  |  |  |  |  | |  |  |  |
| 17 | | interior; likely inaccessible | R113 | | | A117 | | D116 |  |  |  |  | |  |  |  |
| 18 | | interior; likely inaccessible | A110 | | | R113 | | D116 |  |  |  |  | |  |  |  |
| 19 | | exterior; likely accessible | S14 | | | Y72 | | S15 | A16 | W52 | A49 | F46 | |  |  |  |
| 20 | | interior; likely inaccessible | F35 | | | L31 | | Q34 | T118 |  |  |  | |  |  |  |
| **Ivermectin** | | | | | | | | | | | | | | | | |
| **Docking #** | | **Region** | **Residues** | | | | | | | | | | | | | |
| 1 | | interior; likely inaccessible | P78 | | | A82 | | A120 | S86 | D116 | V119 | S124 | | L79 | |  |
| 2 | | exterior; likely accessible | A147 | | | Y12 | | S74 | N73 | G137 | M138 |  | |  | |  |
| 3 | | interior; likely inaccessible | Q46 | | | A49 | | Q45 | L84 | T81 | W52 | A16 | | S14 | | R71 |
| 4 | | interior; likely inaccessible | T89 | | | S86 | | A117 | A120 | A123 | S124 | D116 | | V119 | |  |
| 5 | | exterior; likely accessible | I24 | | | L132 | | V133 | A129 | N126 | N130 | F67 | |  | |  |
| 6 | | interior; likely inaccessible | A40 | | | N33 | | Q47 | L26 | E22 | A51 |  | |  | |  |
| 7 | | exterior; likely accessible | S14 | | | V57 | | T59 | L13 | M58 |  |  | |  | |  |
| 8 | | interior; likely inaccessible | N33 | | | Q47 | | Q46 | L26 |  |  |  | |  | |  |
| 9 | | exterior; likely accessible | S14 | | | V57 | | F10 | V11 | Y72 |  |  | |  | |  |
| 10 | | interior; likely inaccessible | V11 | | | Y72 | | D77 | T81 | A82 | P78 |  | |  | |  |
| 11 | | interior; likely inaccessible | Q46 | | | Q45 | | A40 | L84 | T81 |  |  | |  | |  |
| 12 | | exterior; likely accessible | S8 | | | V11 | | F139 | N73 | P78 |  |  | |  | |  |
| 13 | | inner channel | P102 | | | N97 | | E106 | A101 | P100 | I94 |  | |  | |  |
| 14 | | interior; likely inaccessible | V119 | | | A120 | | N127 | E131 | L79 | P78 |  | |  | |  |
| 15 | | exterior; likely accessible | T28 | | | N25 | | V21 | I24 | F67 |  |  | |  | |  |
| 16 | | exterior; likely accessible | S14 | | | F10 | | V11 | Y72 | S8 | P7 |  | |  | |  |
| 17 | | exterior; likely accessible | S14 | | | S15 | | L13 | F10 |  |  |  | |  | |  |
| 18 | | exterior; likely accessible | D19 | | | K53 | | P54 | V57 |  |  |  | |  | |  |
| 19 | | exterior; likely accessible | N140 | | | F139 | | A142 | G143 | N73 | S74 | T146 | |  | |  |
| 20 | | inner channel | R92 | | | I94 | | I93 | E95 |  |  |  | |  | |  |
| **Rifampicin** | | | | | | | | | | | | | | |  |  |
| **Docking #** | **Region** | | | **Residues** | | | | | | | | | | |  |  |
| 1 | inner channel | | | I107 | N109 | | A110 | | Q112 | R113 |  | |  | |  |  |
| 2 | exterior; likely accessible | | | F139 | A142 | | A147 | | T146 | G137 | T136 | | G135 | |  |  |
| 3 | exterior; likely accessible | | | I24 | F67 | | N140 | | G135 | V133 |  | |  | |  |  |
| 4 | exterior; likely accessible | | | W52 | S15 | | S14 | | R71 | Y72 | V11 | |  | |  |  |
| 5 | exterior; likely accessible | | | P63 | S55 | | V60 | | V57 |  |  | |  | |  |  |
| 6 | interior; likely inaccessible | | | Q46 | D88 | | Q45 | | A49 | N85 | L84 | | S15 | |  |  |
| 7 | exterior; likely accessible | | | G137 | S74 | | N73 | | Y72 | A147 | Y12 | | V11 | |  |  |
| 8 | exterior; likely accessible | | | I24 | L132 | | V133 | | F67 |  |  | |  | |  |  |
| 9 | interior; likely inaccessible | | | D88 | A49 | | Q45 | | N85 | L84 | T81 | |  | |  |  |
| 10 | exterior; likely accessible | | | S15 | S14 | | Y72 | | V11 | F10 |  | |  | |  |  |
| 11 | interior; likely inaccessible | | | A82 | P78 | | L79 | | S124 | N127 | A123 | | A120 | |  |  |
| 12 | interior; likely inaccessible | | | S86 | A82 | | S124 | | L79 |  |  | |  | |  |  |
| 13 | inner channel | | | I107 | V108 | | A110 | | R113 |  |  | |  | |  |  |
| 14 | interior; likely inaccessible | | | D116 | A120 | | V119 | | A123 | S124 |  | |  | |  |  |
| 15 | inner channel | | | E106 | T111 | | Q112 | | D115 |  |  | |  | |  |  |
| 16 | interior; likely inaccessible | | | V11 | Y72 | | D77 | | N73 | S74 |  | |  | |  |  |
| 17 | inner channel | | | I94 | A101 | | N103 | |  |  |  | |  | |  |  |
| 18 | interior; likely inaccessible | | | N85 | S86 | | T89 | | R113 | D116 | A120 | |  | |  |  |
| 19 | interior; likely inaccessible | | | E22 | L26 | | Q47 | | T43 | N29 | N33 | |  | |  |  |
| 20 | exterior; likely accessible | | | S8 | Y72 | | D77 | | N73 |  |  | |  | |  |  |


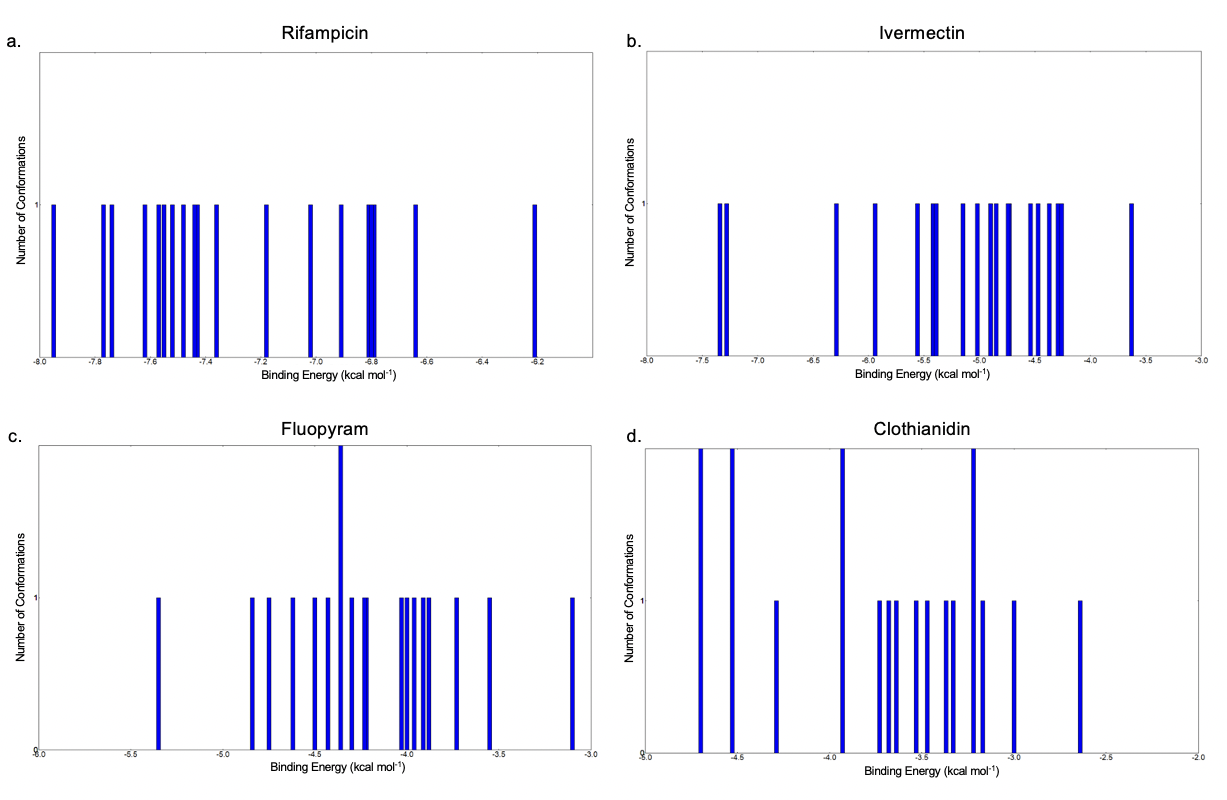


**Figure S6**. The heats of binding (kcal mol^-1^) from the 20 docking models of AIs and TMGMV coat protein (PDB: 1vtm) and number of corresponding conformations at that binding energy. The AIs are rifampicin (a), ivermectin (b), fluopyram (c), and clothianidin (d).
